# Effects of exercise and L-arginine intake on inflammation in aorta of high-fat diet induced obese rats

**DOI:** 10.20463/jenb.2016.03.20.1.6

**Published:** 2016-03-31

**Authors:** Hee-jae Kim, Junseok Son, Eunhee Jin, Jin Lee, Sok Park

**Affiliations:** 1 Physical Activity and Performance Institute (PAPI), Konkuk University, SeoulRepublic of Korea; 2 Health and Exercise Science, Institute of Sport Science, Seoul National University, SeoulRepublic of Korea; 3 Department of Sports Science, Sungkyunkwan University, SuwonRepublic of Korea; 4 Department of Anatomy and Cell Biology, College of Medicine, Han Yang University, SeoulRepublic of Korea; 5 Department of Sports Leadership, Kwangwoon University, SeoulRepublic of Korea

**Keywords:** Exercise, arginine, aorta, inflammation

## Abstract

**[Purpose]:**

In the present study, we investigated the effect of exercise and arginine on the inflammatory makers and Cu-Mn superoxide dismutase (SOD) expression in the aortas of high-fat-induced obese rats.

**[Methods]:**

Fifty 6-month-old male Sprague-Dawley rats were randomly assigned as follows: HF-Con: high-fat diet, HF-Ex: high-fat diet and exercise, HF-Ex+A: high-fat diet and combined exercise and arginine, HF-A: high-fat diet and arginine. The high-fat diet was fed for 12 weeks following 1 week of environmental adaptation with mixed solid chow. The rats performed treadmill exercise 6 times per week for 12 weeks at20 m/min for 60 min. L-argininewas mixed with saline and orally administered at 150 mg/kg once a day. Expressions of inflammatory markers (including NF- κB, TNF-α, COX-2) and SOD were evaluated using western blotting.

**[Results]:**

NF-κB expression decreased significantly (p<0.05) in the HF-Ex group compared with HF-Con group, and we found additional effects(p<0.01) on NF-κB expression in HF-EX+A compared withHF-Ex. TNF-α expression decreased significantly (p<0.01) in HF-Ex, FH-Ex+A, and FH-A compared with HF-Con. In a similar trend with NF-κB expression, COX-2 expression decreased significantly in HF-Ex compared withHF-Con. In Cu-Mn SOD expression, there was no difference between HF and HF-Ex, but significant increases (p<0.01) inCu-Mn SOD werefound in HF-Ex+A and HF-A.

**[Conclusion]:**

Based on our results, treatment that combines exercise and arginine might be effective for modulatingvascular inflammation and oxidative stress in obesity

## INTRODUCTION

Obesity is associated with an increased cardiovascular mortality. Structural and functional changes to thecardiovascularsystem in obesity include ventricular hypertrophy, diastolic dysfunction, and aortic stiffness^1,2^. Virtually all arteries including the aorta are surrounded by significant amounts of perivascular adipose tissue^3^.Regarding cardiovascular risk factors, obesity is associated with increased aortic pulse wave velocity^4^ and predominantly distal patternsof aortic stiffness. According to a review article that discussed obesity and the aorta^5^, obese individuals have excess abdominal visceral fat, which is a better predictor of cardiovascular and metabolic risk than total body fat alone and is also linked to altered vascular function^2^.

L-arginine is an important amino acid and precursor in the biosynthesis of various biologically important compounds such asproteins, nitric oxide (NO), agmatine, creatine, urea, and polyamines^6^. It has been shown that arginine is the only substrate for NO production and that arginine has a crucial effect on the functioning of the cardiovascular system. In the previous studies, arginine exerted aregulatory effect on vascular homeostasis in hypertensive and diabetic patients and in healthy individuals^7,8,9,10^.

Exercise training,in particular aerobic exercise, produced predictable changes in body composition including increased skeletal musculature and decreased fat mass. More importantly, physical activity with endurance training has been known to improve cardiovascular function in human and experimental animals^11,12^. Long-term aerobic exercise markedly improved abnormal hemorheologic properties and oxidative stress in hypercholesterolemicrats. It has been shown that aerobic training positivelyaffects free radicals, lipid peroxides, and the prevention and treatment of cardiovascular disease^13,14^.

However, there is a lack of research on the effects of endurance training on obesity resulting in increased inflammatory responseand antioxidant enzymes. Therefore, we investigated the effectsof exercise and arginine on the inflammatory markers(including NF-κB, TNF-α, COX-2) and Cu-Mn SOD expression in the aortasof high-fat-diet-induced obese rats.

## METHODS

### Experimental animals

Fifty 6-month-old male Sprague-Dawley rats were obtained from Samtako Bio (Osan, Korea) for the experiment. The experiment was performed following 1 week of environmental adaptation and randomization. Eight rats were randomly assigned to one of the following four experimental groups: HF-Con: high-fat diet, HF-Ex: high-fat diet and exercise, HF-EX+A: high-fat diet and combined treatment of exercise and arginine, HF-A: high-fat diet and arginine.

### High fat diet

Rats in the high-fat-diet groups were fed with high-fatchow (Samyang Co., Korea) thatconsisted of 60% fat in total calories. Each experimental animal was cared for with the high-fat diet shown in Table 1 for 12 weeks following the 1-week environmental adaptation with mixed solid chow.

**Table 1.** High-fat diet composition

Ingredient	Content (g/kg)
Casein	200
L-Cystine	3
Maltodextrin	125
Sucrose	68.8
Cellulose	50
Soybean Oil	25
Lard	245
Mineral Mix	10
DiCalcium	13
Phosphate	5.5
Calcium	16.5
Carbonate	10
Potassium Citrate	2
Vitamin Mix	10
Choline Bitartrate	2

### L-arginine administration

The L-arginine (Sigma-Aldrich, St. Louis, MO, USA) administration method in Lee et al.^15^ was applied. L-arginine was mixed with saline and orally administered with 150 mg/kg once a day at the same time for 12 weeks in the HF-A and HF-Ex + Agroups. For HF and EX, the same amount of saline was given via oral administration.

### Exercise protocol

The exercise intervention method of Lee et al.^15^ was applied in this study. The rats performed the exercise 6 times per week for 12 weeks on the rodent treadmill at 0% incline. The initial treadmill speed was set to 15, and 2 m/ min wasadded every 2 weeks to simulate the intensity and effect of exercisetraining. The maximal treadmill speed was limited to 20m/min in the last 2 weeks.

### Tissue preparation and western blotting

After the experimental period, all rats were fasted for 12 hours and anesthetized with a ketamine/xylazine mixture. The aorta was rapidly removed and washed in a phosphatebuffered solution (PBS). Sampled tissues were homogenized in lysis buffer (Cell Signaling Technology, Danvers, MA) with PMSF at 4°C and centrifuged (13,000 x g). The protein content of each sample was determined by the Bradford method (1976)^16^ with bovine serum albumin as a standard. Protein samples (35 μg) were boiled with 5x sample buffer, electrophoresed on polyacrylamide gels, and transferred to a nitrocellulose membrane at 15V overnight. The membrane was washed, blocked, and incubated with antibodies to detect NF-κB, TNF-α, COX-2 (Cell Signaling, Danvers, MA, State, 1:1000), Cu-SOD, and Mn-SOD (1:1500; Chemicon, Temecula, CA, USA) for 12 hr at 4°C. HRP-linked secondary antibody (1:5000; Santa Cruz Biotechonology, Santa Cruz, CA, USA) was added for 1 hr at room temperature. The membranes were washed and visualized by autoradiography after development with an ECL Plus Kit (GEHealthcare Bio-Sciences Crop, Piscataway, NJ, USA). ß-actin was used as an internal control. Densitometry was performed with gel documentation equipment (Gel Doc 2000, Quantity One, Bio-Rad, Hercules, CA, USA).

### Statistical Analysis

All data are expressed as means ±S.E.M. and analyzed by two-way ANOVA (GraphPad software, GraphPad, Santiago, CA, USA) using the procedures in SPSS software (SPSS Inc, 12.0, Chicago, IL) with Bonferroni posttests. p< 0.05 was considered statistically significant.

## RESULTS

**Figure 1.**
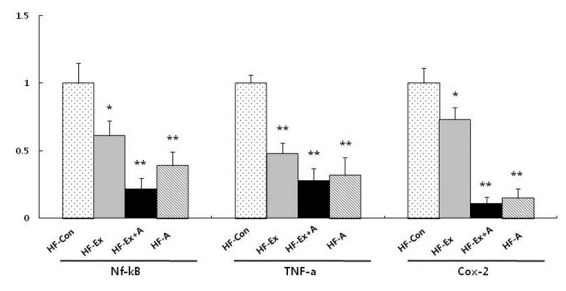
Effectsof exercise and arginine treatment on the expression of inflammation-related factors such as Nf-kB, TNF-a, and COX-2 in high-fatdiet-induced obese rats. Data are presented as mean ±S.E.M. *p<0.05 and **p<0.01 vs. HFCon. HF-Con: high-fat diet, HFEx: high-fat diet and exercise, HF-EX+A: high-fat diet and combined exercise and arginine, HF-A: high-fat diet and arginine.

**Figure 2.**
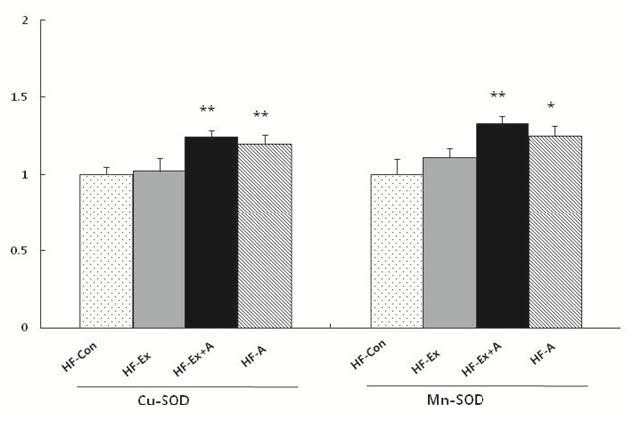
Effectsof exercise and arginine treatment on the expression of Cu-SOD and Mn-SOD in high-fat-diet-induced obese rats. Data are presented as mean ±S.E.M. *p<0.05 and **p<0.01 vs. HF-Con. HF-Con: high-fat diet, HF-Ex: high-fat diet and exercise, HF-EX+A: high-fat diet and combined exercise and arginine, HF-A: high-fat diet and arginine.

Inflammation-related factors including NF-κB, TNF-α, and COX-2 were evaluated after exercise and arginine intervention in obese rats. As shown inFig. 1, NF-κBexpression decreased significantly (p<0.05) in HF-Ex . We also found the most potent effect (p<0.01) on NF-κBexpression in HF-Ex+A. TNF-αexpression decreased significantly (p<0.01) in HF-Ex, FH-Ex+A, and FH-A; however, there was no additional or synergistic effect of combined treatment. Similar toNF-κBexpression, COX-2 expression decreased significantly in HF-Ex. In addition, COX-2 in FH-Ex+A and HF-A showed lower expression than did HFEx. However, there was no significant difference between HF-Ex+A and HF-A (Fig. 1). The expression of the antioxidant enzyme Cu-Mn SOD was measured after exercise and arginine intervention in obese rats. In Cu-SOD expression, there was no difference between HF and HF-Ex, but significant increases (p<0.01) inCu-SOD werefound in HF-Ex+A and HF-A. Similar to Cu-SOD, the expression of Mn-SOD was significantly increased in HF-Ex+A and HF-A but not in HF-Ex (Fig. 2).

## DISCUSSION

In the present study, we found that exercise and arginine treatment significantly decreased inflammatory responses including NF-κB, TNF-α, and COX-2 expressions in the aortasof high-fat-diet-induced obese rats. In addition,-Cu-SOD and Mn-SOD expressions increased significantly in the exercise and argininetreatmentgroup. High-fat diets are generally used by researchers to induce obesity, lipid accumulation, and atherosclerosis vascular disease in rodents17, and studies have shown that a high-fat diet and lack of physical exercise are the most important factors in developing obesity. Therefore, based on our results, exercise and arginine treatment might have positive effects on inflammatory response and antioxidant enzyme expression.

Chronic vascular inflammation is afundamental mechanism invascular diseases associated with variety of risk factors, contributing to the pathogenesis of atherosclerosis and plaque rupture and leading to acute coronary syndrome^34^. At the cellular and molecular levels, oxidative stress, vascular inflammation, and endothelial cell dysfunction can result, which is mainly reflected vasoprotective endothelial NO bioavailability^31^. According to a recent review article^32^, the role of inflammation and the underlying mechanismsin atherogenesis and atherothrombosis are comprehensively reviewed and discussed by many reports. In addition, a number of recent high-profile reports have demonstrated the requirement for components of the NF-κB signaling apparatus in developing metabolic disease including obesity ^36^.

Nitric oxide, a product of arginine, is generated by the three isomorphic forms of nitric oxide synthase (NOS): neuronal NOS (nNOS), inducible NOS (iNOS) and endothelial NOS (eNOS), which are widely expressed in virtually all vascular cell types^18,19,20^. NO influences a number of metabolic, biosynthetic, signaling, and membrane transport processes^19,21^, and one of itsimportant roles is regulating vascular tone and structure^19,22^. In previous reports, acute and chronic arginine treatment improve endothelial function in hypercholesterolemia and atherosclerosis models^23^. Increased plasma arginine concentration leads to increased production of both vascular and systemic NO^24,25,26^. Stimulated NO production leads to relaxed vascular smooth muscle cells^19,27^,and reduced bioavailability of NO as a result of either decreased NOS production or increased breakdown by reactive oxygen species (ROS) is implicated in the development of various vascular disorders^28,29,30^.

Oxidative stress is characterized bythe excess production of oxidant molecules that overwhelm the antioxidant defense systems, resulting in oxidative damage^31^. Multiple enzymes involved in oxidative stress within the vascular wall can be stimulated or up-regulated in the presence of cardiovascular risk factors, leading to excess ROS production and cellular damage^31,32^. In addition, superoxide anion (O2-) is the parent ROS molecule produced by one electron reduction of oxygen catabolized by various enzymes including cycolooxygenase, lipoxygenases, and cytochrome P459 enzymes^33^. In addition, SOD and glutathione peroxidase activity in individuals with obesity is significantly lower compared with that in healthy persons, having implications for the development of obesity-related health problems^35^.

In our study limitation, we did not measure and evaluate inflammatory responses such as pro-inflammatory cytokines and chemokines in circulation, and it is necessary to evaluate the changes in vascular structure such as vascular fibrosis and stiffness that are affected by obesity or combined exercise and arginine treatment. It might be more powerful to measure the other antioxidant enzymes such as catalase and glutathion peroxidase. Furthermore, enzyme activity must also be investigated in addition to enzyme expressionfor the better understanding of substrate utilization.

In the present study, exercise and argininetreatment significantly attenuatedthe inflammatory response with increasedantioxidantenzymes and Cu-Mn SOD expression in the aortas of high-fat-diet-induced obese rats. Based on the previous reports and our results, combined exercise and arginine treatment might be effective for modulatingvascular inflammation and oxidative stress in obesity. Future studies should identify the optimal exercise intensity or duration considering arginine dose for treating obesity-induced vascular dysfunction.

## References

[1] Alpert MA. (2001). Obesity cardiomyopathy: pathophysiology and evolution of the clinical syndrome. Am J Med Sci.

[2] Sutton-Tyrrell K., Newman A., Simonsick EM., Havlik R., Pahor M., Lakatta E. (2001). Aortic stiffness is associated with visceral adiposity in older adults enrolled in the study of health, aging, and body composition. Hypertension.

[3] Police SB., Thatcher SE., Charnigo R., Daugherty A., Cassis LA. (2009). Obesity promotes inflammation in periaortic adipose tissue and angiotensin II-induced abdominal aortic aneurysm formation. Arterioscler Thromb Vasc Biol.

[4] Rider OJ., Tayal U., Francis JM., Ali MK., Robinson MR., Byrne JP. (2010). The effect of obesity and weight loss on aortic pulse wave velocity as assessed by magnetic resonance imaging. Obesity (Silver Spring).

[5] Rider OJ., Lewis AJ., Neubauer S. (2014). Structural and Metabolic Effects of Obesity on the Myocardium and the Aorta.. Obes facts.

[6] Milovanovic ES., Obradovic M., Jovanovic A., Zaric B., Zafirovic S., Panic A. (2015). Benefits of L-Arginine on cardiovascular system. Mini Rev Med Chem.

[7] Cylwik D., Mogielnicki A., Buczko W. (2005). L-arginine and cardiovascular system. Pharmacol Rep.

[8] Giugliano D., Marfella R., Verrazzo G., Acampora R., Nappo F., Ziccardi P. (1997). L-arginine for testing endothelium-dependent vascular functions in health and disease. Am J Physiol.

[9] Gryglewski RJ., Grodzinska L., Kostka-Trabka E., Korbut R., Bieroon K., Goszcz A. (1996). Treatment with L-arginine is likely to stimulate generation of nitric oxide in patients with peripheral arterial obstructive disease. Wien Klin Wochenschr.

[10] Stief TW., Weippert M., Kretschmer V., Renz H. (2001). Arginine inhibits hemostasis activation. Thromb Res.

[11] Deley G., Kervio G., Van Hoecke J., Verges B., Grassi B., Casillas JM. (2007). Effects of a one-year exercise training program in adults over 70 years old: a study with a control group. Aging Clin Exp Res.

[12] Lee J., Cho HS., Park S., Kim WK. (2009). Regular exercise produced cardioprotective effects on rat’s heart with hypertension induced by L-NAME administration. Clin Exp Hypertens.

[13] Hambrecht R., Adams V., Erbs S., Linke A., Krankel N., Shu Y. (2003). Regular physical activity improves endothelial function in patients with coronary artery disease by increasing phosphorylation of endothelial nitric oxide synthase. Circulation.

[14] Jia B., Wang X., Kang A., Wang X., Wen Z., Yao W. (2012). The effects of long term aerobic exercise on the hemorheology in rats fed with high-fat diet. Clin Hemorheol Microcirc.

[15] Kim SY., Lee J. (2014). Exercise Training suppresses vascular fibrosis in aging obesity induced rats. J Exerc Nutrition Biochem.

[16] Bradford MM. (1976). A rapid and sensitive method for the quantitation of microgram quantities of protein utilizing the principle of protein-dye binding. Anal Biochem.

[17] Quan Y., Qian MZ. (2010). Effect and mechanism of gypenoside on the inflammatory molecular expression in high-fat induced atherosclerosis rats. Zhongguo Zhong Xi Yi Jie He Za Zhi.

[18] Kypreos KE., Zafirovic S., Petropoulou PI., Bjelogrlic P., Resanovic I., Traish A. (2014). Regulation of endothelial nitric oxide synthase and high-density lipoprotein quality by estradiol in cardiovascular pathology. J Cardiovasc Pharmacol Ther.

[19] Dobutović B., Smiljanić K., Soskić S., Düngen H-D., Isenović ER. (2011). Nitric Oxide and its Role in Cardiovascular Diseases. Open Nitric Oxide J.

[20] Grisham MB., Jourd’Heuil D., Wink DA. (1999). Nitric oxide. I. Physiological chemistry of nitric oxide and its metabolites:implications in inflammation. Am J Physiol.

[21] Mayer B., Hemmens B. (1997). Biosynthesis and action of nitric oxide in mammalian cells. Trends Biochem Sci.

[22] Dalsgaard T., Kroigaard C., Simonsen U. (2010). Calcium-activated potassium channels - a therapeutic target for modulating nitric oxide in cardiovascular disease?. Expert Opin Ther Targets.

[23] Bode-Boger SM., Scalera F., Ignarro LJ. (2007). The L-arginine paradox: Importance of the L-arginine/asymmetrical dimethylarginine ratio. J Pharmacol Exp Ther.

[24] Wu G. (1998). Intestinal mucosal amino acid catabolism. J Nutr.

[25] Tousoulis D., Antoniades C., Tentolouris C., Goumas G., Stefanadis C., Toutouzas P. (2002). L-arginine in cardiovascular disease: dream or reality?. Vasc Med.

[26] Maxwell MJ., Cooke JP. (1998). Cardiovascular effects of L-arginine. Curr Opin Nephrol Hypertens.

[27] Palmer RM., Ashton DS., Moncada S. (1988). Vascular endothelial cells synthesize nitric oxide from L-arginine. Nature.

[28] Naseem KM. (2005). The role of nitric oxide in cardiovascular diseases. Mol Aspects Med.

[29] Napoli C., Ignarro LJ. (2009). Nitric oxide and pathogenic mechanisms J Exerc Nutrition Biochem. 2016;20(1):xxx-xxx, http://dx.doi.org/10.20463/jenb.2016.03.20.1.6 5 Exercise and arginine in vascular inflammation Journal of Exercise Nutrition & Biochemistry involved in the development of vascular diseases. Arch Pharm Res.

[30] Zago AS., Zanesco A. (2006). Nitric oxide, cardiovascular disease and physical exercise. Arq Bras Cardiol.

[31] Lonn ME., Dennis JM., Stocker R. (2012). Actions of “antioxidants” in the protection against atherosclerosis. Free Radic Biol Med.

[32] Yang Z., Ming XF. (2013). Arginase: the emerging therapeutic target for vascular oxidative stress and inflammation. Front Immunol.

[33] Yang Z., Lüscher TF., Lanzer P., Topol EJ. (2002). Vascular Endothelium.

[34] Hansson GK., Hermansson A. (2011). The immune system in atherosclerosis. Nat Immunol.

[35] Ozata M., Mergen M., Oktenli C., Aydin A., Sanisoglu S., Bolu E., Yilmaz M., Sayal A., Isimer A., Ozdemir I. (2002). Increased oxdative stress and hypozincemia in male obesity. Clin Biochem.

[36] Baker RG., Hayden MS., Shosh S. (2011). NF-κB, inflammation, and metabolic disease. Cell Metab.

